# The Paraventricular Nucleus of the Thalamus as an Integrating and Relay Node in the Brain Anxiety Network

**DOI:** 10.3389/fnbeh.2021.627633

**Published:** 2021-02-24

**Authors:** Gilbert J. Kirouac

**Affiliations:** Department of Oral Biology, Dr. Gerald Niznick College of Dentistry, Rady Faculty of Health Sciences, University of Manitoba, Winnipeg, MB, Canada

**Keywords:** anxiety, paraventricular nucleus, thalamus, stress, nucleus accumbens, extended amygdala

## Abstract

The brain anxiety network is composed of a number of interconnected cortical regions that detect threats and execute appropriate defensive responses via projections to the shell of the nucleus accumbens (NAcSh), dorsolateral region of the bed nucleus of the stria terminalis (BSTDL) and lateral region of the central nucleus of the amygdala (CeL). The paraventricular nucleus of the thalamus (PVT) is anatomically positioned to integrate threat- and arousal-related signals from cortex and hypothalamus and then relay these signals to neural circuits in the NAcSh, BSTDL, and CeL that mediate defensive responses. This review describes the anatomical connections of the PVT that support the view that the PVT may be a critical node in the brain anxiety network. Experimental findings are reviewed showing that the arousal peptides orexins (hypocretins) act at the PVT to promote avoidance of potential threats especially following exposure of rats to a single episode of footshocks. Recent anatomical and experimental findings are discussed which show that neurons in the PVT provide divergent projections to subcortical regions that mediate defensive behaviors and that the projection to the NAcSh is critical for the enhanced social avoidance displayed in rats exposed to footshocks. A theoretical model is proposed for how the PVT integrates cortical and hypothalamic signals to modulate the behavioral responses associated with anxiety and other challenging situations.

## Introduction

Anxiety is an ethologically advantageous emotion that maximizes survival because it promotes avoidance of potential harm in situations where dangers can emerge quickly (Steimer, 2002; Calhoon and Tye, 2015; LeDoux and Daw, 2018). It is characterized by a state of arousal and hypervigilance in addition to excessive behavioral avoidance of potential threats. Unnecessary levels of anxiety can cause significant distress and a better understanding of how neural circuits in the brain control responses to threats and mediate anxiety is considered important for the development of new treatments (Craske et al., 2017). It is generally accepted that anxiety results from genetic vulnerabilities combined with situational factors like stress and exposure to fear-inducing situations (Nemeroff et al., 2006; Craske and Stein, 2016). There are a number of comprehensive reviews describing the components of the anxiety network (Adhikari, 2014; Calhoon and Tye, 2015; Tovote et al., 2015; LeDoux and Daw, 2018). The network involves a number of interconnected cortical and subcortical regions that evaluate and respond to potential threats (Steimer, 2002; Calhoon and Tye, 2015; LeDoux and Daw, 2018). How these regions function together as a network is poorly understood and is of considerable interest from preclinical and clinical perspectives (Shin and Liberzon, 2010; Adhikari, 2014; Calhoon and Tye, 2015; Fox and Shackman, 2017; Silva and McNaughton, 2019).

The present review presents evidence that the paraventricular nucleus of the thalamus (PVT) may be a critical node in the brain anxiety network. Anatomical details of how the PVT is connected with many components of the brain’s anxiety network are presented as well as recent evidence showing that neurotransmission to the PVT from orexin neurons in the hypothalamus contributes to stress-induced anxiety. The review also discusses recent anatomical evidence that shows that neurons in the PVT provide divergent projections to key striatal-like subcortical regions involved in the various defensive responses. Finally, a model is described that postulates that the PVT integrates and amplifies cortical signals related to threats and relays these signals to activate subcortical circuits that modulate defensive responses including avoidance of potential threats.

## Stress and Anxiety

Fear and anxiety are similar types of emotions (LeDoux, 2015; LeDoux and Pine, 2016) that can be inferred in experimental animals from the expression of stereotypical defensive behaviors (LeDoux, 2015; LeDoux and Pine, 2016). Fear is triggered by the presence of an *impending threat* and is often experimentally defined in rodents as freezing to conditioned cues or contexts. In contrast, anxiety is a response to *potential threats* and is operationally defined as avoidance of potential risks involving open spaces, bright lights, and novel conspecifics (Steimer, 2002; LeDoux, 2015). An association between stress and anxiety is well-established (Shin and Liberzon, 2010; Bystritsky and Kronemyer, 2014; Daviu et al., 2019) with stress being a contributing factor for most anxiety disorders including social anxiety disorder and posttraumatic stress disorder (PTSD) (Bystritsky and Kronemyer, 2014; Carvajal, 2018). The causal relationship between stress and anxiety is most dramatically exemplified by PTSD where a single but intensely stressful experience can lead to a long-lasting anxiety state in susceptible individuals (Nemeroff et al., 2006; Craske and Stein, 2016). The enduring effect of acute stress on behavior is not unique to humans since exposure of rodents to a single episode of footshocks produces enhanced levels of anxiety, including heightened level of social avoidance that does not appear to be directly dependent of the retrieval of a fear memory (Louvart et al., 2005; Siegmund and Wotjak, 2007; Mikics et al., 2008; Chen et al., 2012).

## Brief Overview of the PVT and Its Role in Regulating Behavior

The PVT has received a considerable amount of attention because of its connections with regions of the brain linked to the regulation of emotional and motivated behavior (Groenewegen et al., 1987; Berendse and Groenewegen, 1990, 1991; Berendse et al., 1992; Wright and Groenewegen, 1995, 1996). Tracing studies show that the PVT sends a robust excitatory projection to a continuum in the basal forebrain that includes the shell of the nucleus accumbens (NAcSh), dorsolateral region of the bed nucleus of the stria terminalis (BSTDL) and lateral region of the central nucleus of the amygdala (CeL) consisting of the lateral and capsular subnuclei of the central nucleus of the amygdala (Moga et al., 1995; Parsons et al., 2007; Li and Kirouac, 2008; Vertes and Hoover, 2008; Dong et al., 2017). The BSTDL and CeL are part of a larger macrostructure called the central extended amygdala (cEA) (Heimer and Alheid, 1991; Alheid et al., 1995; de Olmos et al., 2004). The cEA contains striatal-like projection neurons that send fibers to the hypothalamus and brainstem. In addition, the cEA densely innervates the medial extended amygdala (mEA), which consists of the medial regions of the bed nucleus of the stria terminalis and the medial central nucleus of the amygdala (Heimer and Alheid, 1991; Alheid et al., 1995; de Olmos et al., 2004). The mEA is composed of pallidal-like neurons that provide dense descending projections to the somatomotor, visceromotor, and endocrine circuits in the hypothalamus and brainstem known for producing some of the physiological and behavioral responses that make up defensive responses (Petrovich and Swanson, 1997; Cassell et al., 1999; Swanson, 2000). The NAcSh of the ventral striatum is composed of medium spiny neurons that provide fiber projections to the ventral pallidum, lateral hypothalamus, and ventral tegmental area (Heimer et al., 1991; Zahm and Brog, 1992; Zahm, 2006). While the NAcSh is sometimes considered a transitional region between the cEA and the rest of the striatum, it is also appropriate to consider the NAcSh, BSTDL, and CeL as components of a large striatal-like descending macrosystem involved in the regulation of complex behavior (Swanson, 2000; Zahm, 2006). A notable common anatomical feature of this striatal-like macrosystem is an exceptionally dense plexus of PVT fibers (Li and Kirouac, 2008). It is also important to appreciate that PVT neurons project weakly to cortical regions (i.e., prelimbic, infralimbic, anterior insular cortices; ventral subiculum, and the basolateral nucleus of the amygdala) that innervate the same areas of the NAcSh, BSTDL, and CeL that receive fibers from the PVT (Kirouac, 2015). This places the PVT in a position to influence multiple levels of the cortico-subcotical circuits involved in regulating behavior.

The sources of input to the PVT have also been examined (Otake et al., 1995, 2002; Krout and Loewy, 2000a, b; Krout et al., 2002) and a detailed comparative analysis of all afferents to the PVT using retrograde tracing methods indicate that the major sources of inputs originate from neurons in the prefrontal cortex (infralimbic, prelimbic, insular) and the ventral subiculum of the hippocampus (Li and Kirouac, 2012). The robustness of cortical projections to the PVT has also been described using anterograde tracing methods (Sesack et al., 1989; Canteras and Swanson, 1992; Vertes, 2004). In addition, a number of functionally distinct nuclei in the brainstem and hypothalamus known to modulate behavioral states are also significant sources of afferents to the PVT (Otake et al., 1995, 2002; Krout and Loewy, 2000a, b; Krout et al., 2002), but the strength of these inputs often appear to be eclipsed by the comparative strength of the cortical inputs (Li and Kirouac, 2012).

It is also notable that the PVT is an area of the brain consistently identified as being activated during states of behavioral arousal (reviewed in Kirouac, 2015; Millan et al., 2017; Barson et al., 2020; McGinty and Otis, 2020) including those where sensory cues predict rewarding or aversive conditions (Do-Monte et al., 2015; Zhu et al., 2018; Choi et al., 2019). Recording of calcium signals or single unit activity in the PVT of behaving animals exposed to stimuli associated with appetitive or aversive outcomes indicates that PVT neurons respond robustly to novel cues and that these neurons track the saliency of these cues (Do-Monte et al., 2015, 2017; Choi and McNally, 2017; Zhu et al., 2018; Otis et al., 2019). A considerable amount of direct experimental evidence is also available demonstrating that the PVT contributes to conditioned appetitive and defensive behaviors in a projection specific manner (Do-Monte et al., 2015, 2017; Labouebe et al., 2016; Zhu et al., 2016, 2018; Choi and McNally, 2017; Cheng et al., 2018; Choi et al., 2019). The critical question of whether the PVT preferentially promotes appetitive or aversive responses has not been unequivocally resolved (some of the controversies and challenges in studying the role of the PVT in behavior was recently reviewed in McGinty and Otis, 2020). Indeed, the type of influence the PVT has on behavior studied in a laboratory setting may be in part dependent on whether opto- or chemogenetic methods are targeted at a specific projection system. For instance, some studies have shown that PVT neurons that project to the CeL contribute to the behavioral freezing associated with conditioned fear (Do-Monte et al., 2015; Penzo et al., 2015) while others have shown that PVT neurons that project to the NAcSh mediate conditioned sucrose seeking (Labouebe et al., 2016; Cheng et al., 2018). This suggests that PVT neurons that innervate the CeL mediate aversive responses while those that project to the NAcSh mediate appetitive responses. However, this is not supported by other evidence showing that PVT neurons that project to the NAcSh mediate real-time avoidance and conditioned place avoidance (Zhu et al., 2016). It should be clear that our understanding of how the PVT mediates valence-dependent responses is incomplete and is further complicated by the fact that many neurons in the PVT send bifurcating axons that innervate multiple target areas of the forebrain (Unzai et al., 2015; Dong et al., 2017). Divergence of projections from single neurons in the PVT implies that PVT neurons may coordinate behavioral responses by simultaneously engaging multiple subcortical circuits. Furthermore, more recent evidence indicates that the type of influence the PVT has on behavior may be dependent on the type of experimental paradigm being studied and whether competing motivational states are present (Choi and McNally, 2017; Do-Monte et al., 2017; Choi et al., 2019; McGinty and Otis, 2020). Some of the evidence and potential mechanisms by which the PVT influences motivated behavior have been discussed in recent reviews and will not be considered in all of their intricate details here (Kirouac, 2015; Do Monte et al., 2016; Millan et al., 2017). While all neurons in the PVT are presumed to be projection neurons that use excitatory amino acids as their main neurotransmitter (Christie et al., 1987; Frassoni et al., 1997), it is also important to appreciate that the PVT is not a uniform structure (Kirouac, 2015). For example, the anterior (aPVT) and posterior aspect of the PVT (pPVT) are composed of neurons that have preferential efferent targets and different sources of afferent inputs making these two broad regions of the PVT potentially functionally different (Li and Kirouac, 2008, 2012; Kirouac, 2015; Dong et al., 2019).

## The Brain Anxiety Network

Anxiety states and avoidance of threats are regulated by brain circuits engaged in hierarchical control of defensive strategies in what has been conceptualized as the brain anxiety network (Adhikari, 2014; Calhoon and Tye, 2015; Tovote et al., 2015; LeDoux and Daw, 2018). Potential threats are detected through coordinated activity in a network of interconnected cortical areas that include the basolateral nucleus of the amygdala, ventral hippocampus, and prefrontal cortex (Adhikari, 2014; Calhoon and Tye, 2015; Silva and McNaughton, 2019). It is postulated that this cortical network evaluates potential risks and initiates defensive responses via projections to the subcortical regions associated with the selection of behavioral responses (Kim et al., 2013; Adhikari, 2014; Duvarci and Pare, 2014; Calhoon and Tye, 2015; Fox et al., 2015; Fox and Shackman, 2017). The BSTDL is the part of the cEA that has been most studied for its role in anxiety (Walker et al., 2003, 2009; Kim et al., 2013; Pleil et al., 2015; Normandeau et al., 2018). Optogenetic activation of the BSTDL elicited anxiety in the elevated plus maze (EPM), whereas inhibition has an anxiolytic effect (Kim et al., 2013). The neural connections by which the BSTDL modulates anxiety appear to involve connections to other regions of the bed nucleus of the stria terminalis (i.e., mEA) which exert modulatory effects on anxiety via descending projections to the lateral hypothalamus and ventral tegmental area (Jennings et al., 2013; Kim et al., 2013). The CeL has been primarily investigated for its role in conditioned fear (Kalin et al., 2004; Cai et al., 2012; Ventura-Silva et al., 2013), but recent evidence indicates that the CeL also potentially contributes to anxiety via projections to the BSTDL (Ahrens et al., 2018; Asok et al., 2018a). The NAcSh has been mostly studied for its role in reward and appetitive behaviors (Nicola, 2007; Floresco, 2015; Namburi et al., 2016). However, there is ample evidence that the NAcSh regulates defensive responses (Reynolds and Berridge, 2001, 2002, 2003, 2008; Newton et al., 2002; Barrot et al., 2005; Al-Hasani et al., 2015; Ramirez et al., 2015; Zhu et al., 2016; Anderson et al., 2018; Lee et al., 2018; Piantadosi et al., 2018) including anxiety (Martinez et al., 2002; Lopes et al., 2007, 2012; da Cunha et al., 2008). It is generally accepted that the striatum including the NAcSh integrates cognitive and affective information from the cortex and thalamus in a way that leads to the selection or promotion of an appropriate behavioral response (Nicola, 2007; Floresco, 2015; Namburi et al., 2016) especially where the outcome of an action is ambiguous (Namburi et al., 2016). From this perspective, the NAcSh may contribute to avoidance by biasing motivational/emotional responses in the defensive direction in situations involving both benefits and threats (i.e., approach-avoidance conflicts). The NAcSh is also emerging as an area of the brain critical for social interaction where disruption of normal signaling contributes to social avoidance (Aragona et al., 2006; Christoffel et al., 2011; Chaudhury et al., 2013; Dolen et al., 2013; Gunaydin et al., 2014; Francis et al., 2015; Resendez et al., 2016; Wook Koo et al., 2016; Rademacher et al., 2017; Folkes et al., 2019; Steinman et al., 2019). Our understanding of the neural mechanisms by which the NAcSh mediates the avoidance induced by anxiety is incomplete but is likely to involve projections to the ventral tegmental area and the lateral hypothalamus (Zahm and Brog, 1992; Zahm, 1999, 2000, 2006; da Cunha et al., 2008; Al-Hasani et al., 2015; Zhu et al., 2016) similar to how the BSTDL modulates anxiety-like responses (Jennings et al., 2013; Kim et al., 2013).

## Anatomical Connections Between the PVT and the Anxiety Network

**FIGURE 1:**
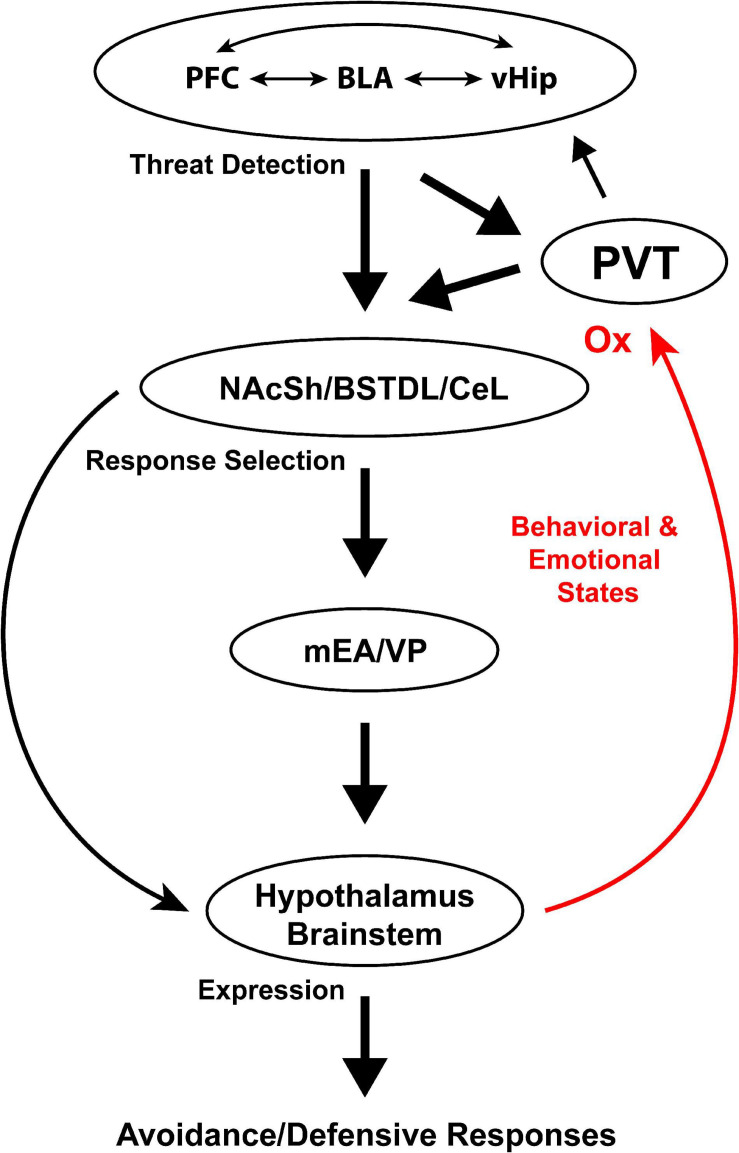
Anatomical connections of the PVT with components of the anxiety network. BLA, basolateral amygdala; BSTDL, dorsolateral region of the bed nucleus of the stria terminalis; CeL, lateral region of the central nucleus of the amygdala; LH, lateral hypothalamus; mEA, medial extended amygdala; NAcSh, shell of the nucleus accumbens; OX, orexins; PFC, prefrontal cortex; PVT, paraventricular nucleus of the thalamus; vHip, ventral hippocampus; VP, ventral pallidum; VTA, ventral tegmental area.

Figure 1 illustrates the most prominent connections between the PVT and components of the anxiety network. As shown, the PVT is well-positioned to contribute to the selection of defensive responses via a dense projection to the NAcSh, BSTDL, and CeL (Moga et al., 1995; Parsons et al., 2007; Li and Kirouac, 2008; Vertes and Hoover, 2008; Dong et al., 2017). These striatal-like regions are in turn anatomically positioned to modulate defensive circuits in the mEA, hypothalamus and brainstem. The PVT is also suitably placed to relay arousal related signals to the cortical network involved in threat detection. In addition to being connected with the critical output components of the anxiety network, the PVT is anatomically positioned to integrate and relay a variety of signals known to contribute to anxiety. For example, the prelimbic cortex is a major source of input to the PVT (Sesack et al., 1989; Canteras and Swanson, 1992; Vertes, 2004; Li and Kirouac, 2012) and experimental evidence indicates that the prelimbic cortex not only promotes conditioned fear responses but also contributes to fear generalization and anxiety (Jinks and McGregor, 1997; Lacroix et al., 2000; Sullivan and Gratton, 2002; Shah and Treit, 2003, 2004; Adhikari et al., 2010, 2011; Lisboa et al., 2010; Sotres-Bayon and Quirk, 2010; Xu and Sudhof, 2013; Rozeske et al., 2015; Yamada et al., 2015; Suzuki et al., 2016; Shimizu et al., 2018). It is especially notable that a projection from the prelimbic cortex to the PVT is critical for retrieval of remote fear memories (Padilla-Coreano et al., 2012; Do-Monte et al., 2015; Do Monte et al., 2016). The significance of the latter findings is that fear memories generalize over time in a way that is believe to contribute to the development of PTSD (Liberzon and Abelson, 2016; Asok et al., 2018b). Consequently, the prelimbic cortex may not only relay signals to the PVT directly associated with a previously experienced threat but also any cues that remotely resemble those present at the time of a fear inducing event. The PVT also receives strong input from the infralimbic cortex, insular cortex and the ventral subiculum (Li and Kirouac, 2012), which have been implicated in stress, anxiety and fear generalization (O’Mara et al., 2009; Adhikari et al., 2010; Bi et al., 2013; Berg et al., 2019; Shi et al., 2020).

The PVT receives afferents from many regions of the hypothalamus including a significant input from the dorsomedial nucleus of the hypothalamus (Li and Kirouac, 2012). The dorsomedial nucleus of the hypothalamus has been shown to contribute to arousal, stress and anxiety (Fontes et al., 2011; Johnson and Shekhar, 2012) and signals from this region of the hypothalamus to the PVT may be critical for promoting anxiety. In addition, retrograde tracing studies have reported that the PVT receives afferent input from neurons scattered in numerous regions of the brainstem (Krout and Loewy, 2000a, b; Krout et al., 2002). In our comparative analysis of all sources of input to the PVT, regions of the brainstem associated with viscerosensory and motor functions including the periaqueductal gray and parabrachial nucleus were found to be the most prominent source of input to the PVT, whereas adrenergic cell groups including those found in the locus ceruleus and other regions of the brainstem were found to be comparatively minor sources of afferents to the PVT (Kirouac et al., 2006; Li and Kirouac, 2012; Li et al., 2014a). The PVT also receives a relatively weak dopaminergic projection that originates from neurons scattered in the hypothalamus and the ventrolateral periaqueductal gray but not the ventral tegmental area (Li et al., 2014a). It is notable that the PVT receives afferents from neurons scattered in a variety of regions in the hypothalamus and brainstem that can be broadly described as having functions related to the modulation of behavioral states in addition to the relay of viscerosensory and nociceptive information (Li and Kirouac, 2012). It is also of interest that many of the neurons in the brainstem and hypothalamus that innervate the PVT produce neuropeptides that may be involved in signaling states of arousal and stress (Freedman and Cassell, 1991; Battaglia et al., 1992; Otake and Nakamura, 1995; Haskell-Luevano et al., 1999; Kirouac et al., 2005, 2006; Otake, 2005; Clark et al., 2011; Hermes et al., 2013; Lee et al., 2015). For example, orexin peptides have been shown to be involved in the regulation of behavioral states (Peyron et al., 1998; Berridge et al., 2010; Boutrel et al., 2010) including those involving aversive and stressful events (Ida et al., 2000; Zhu et al., 2002; Espana et al., 2003; Winsky-Sommerer et al., 2004; Furlong et al., 2009). As previously reviewed, the bottom-up projections to the PVT are postulated to form an ascending emotional arousal system (van der Werf et al., 2002; Kirouac, 2015).

## The PVT as an Emotional Arousal and Stress Responsive Region

Neurons that innervate the PVT originate from regions of the brain that have functions that can be broadly defined as being involved in mediating behavioral or emotional states. For instance, the parabrachial nucleus is a major ascending relay center for viscerosensory and nociceptive information from the body to the forebrain including the PVT (Saper and Loewy, 1980; Gauriau and Bernard, 2002). The hypothalamic inputs originate from neurons located in many regions of the hypothalamus with many of these neurons producing neuropeptides linked to a variety of functions including food intake, arousal, and stress (Freedman and Cassell, 1994; Li and Kirouac, 2012; Colavito et al., 2015; Kirouac, 2015). This suggests that the PVT integrates signals related to a variety of behavioral states including those involved in physiological and psychological challenges. Indeed, many studies have reported that the PVT is activated by exposure of rats to stressful or aversive conditions including restraint (Cullinan et al., 1995; Bhatnagar and Dallman, 1998), tail pinch and footshocks (Smith et al., 1997; Bubser and Deutch, 1999; Yasoshima et al., 2007; Baisley et al., 2011), swimming stress (Cullinan et al., 1995; Zhu et al., 2011), predator scent (Baisley et al., 2011), ultrasonic vocalizations in the dysphoric range (Beckett et al., 1997), aversive visceral stimulation (Yasoshima et al., 2007), and exposure to a context and cues associated with aversive experiences (Beck and Fibiger, 1995; Yasoshima et al., 2007; Padilla-Coreano et al., 2012). Stress-induced activation of the PVT appears to have functional implications since a number of studies have shown that the PVT modulates the neuroendocrine and behavioral responses to chronic stress (Bhatnagar and Dallman, 1998; Bhatnagar et al., 2000, 2002). For example, the pPVT has been shown to be necessary for both the habituation and facilitation of the hypothalamic pituitary axis (HPA) to chronic stress (Bhatnagar and Dallman, 1998, 1999; Bhatnagar et al., 2000, 2002). The HPA response may be facilitated or enhanced in a way that promotes anxiety when an organism encounters novel challenges. In addition, the PVT has been shown to contribute to learned behavioral responses of rodents exposed to aversive and stressful conditions (Li et al., 2011; Padilla-Coreano et al., 2012; Do-Monte et al., 2015; Zhu et al., 2016). More recent evidence indicates that motivational conflicts having both appetitive and aversive consequences selectively activate projection specific neurons in the PVT in a way that promotes a unique behavioral response (Choi and McNally, 2017; Choi et al., 2019). Furthermore, the frustration effect of sucrose reward omission produces a change in how neurons in the PVT that project to the NAcSh or CeL subsequently influence further sucrose seeking (Do-Monte et al., 2017). In summary, the PVT represents a brain region that is generally active during states of high arousal including stressful and aversive conditions as well as the cues previously associated with these conditions (Kirouac, 2015; Do Monte et al., 2016; Barson et al., 2020). This places the PVT in a position to integrate and relay threat- and stress-related signals to the NAcSh and cEA where modulation of local circuits may be involved in the selection of appropriate defensive responses via descending projections. Depending on the situation and proximity of a potential threat, this could involve ceasing normal behavioral activity including all appetitive behaviors, completely stopping all movement (freezing), moving away, or hiding from the perceived threat (Steimer, 2002; Calhoon and Tye, 2015; LeDoux and Daw, 2018).

## Orexin Neurotransmission to the PVT and Stress-Induced Anxiety

The orexin (hypocretin) peptides are exclusively found in neurons of the lateral and perifornical region of the posterior hypothalamus (Peyron et al., 1998; Sakurai et al., 1998). The bioactive orexin-A (OXA) and orexin-B (OXB) peptides are produced by the cleavage of prepro-orexin (ppOX). Orexins act at G protein-coupled receptors called the orexin-1 receptor (OX1R), which is selective for OXA, and the orexin-2 receptor (OX2R), which is non-selective for OXA and OXB (Sakurai et al., 1998; de Lecea, 2012). Orexin neurons have widespread projections to regions of the brain that regulate arousal and behavioral states (Peyron et al., 1998; Berridge et al., 2010; Boutrel et al., 2010). The PVT contains an especially impressive plexus of orexin fibers (Kirouac et al., 2005, 2006) where orexins act to promote wakefulness (Ren et al., 2018); drug (Matzeu et al., 2014) and food reward seeking (Choi et al., 2010; Meffre et al., 2019); and attribution of salience to reward cues (Haight et al., 2020). Orexin neurons become active when animals are exposed to aversive conditions (Ida et al., 2000; Zhu et al., 2002; Espana et al., 2003; Winsky-Sommerer et al., 2004; Furlong et al., 2009) and experimental evidence indicates that orexins modulate the physiological, hormonal and behavioral responses to stress via action in the brain (Kayaba et al., 2003; Furlong et al., 2009; Zhang et al., 2010; Heydendael et al., 2011; Grafe and Bhatnagar, 2018).

Experimental evidence indicates that orexins are involved in fear and anxiety. For instance, systemic administration of a non-specific orexin receptor antagonist reduces fear potentiated startle and the increases in heart rate and blood pressure that are produced when rats are placed in the context previously associated with footshocks (Furlong et al., 2009; Steiner et al., 2012). Research by our own group found that the level of ppOX mRNA is increased in the hypothalamus of rats that developed anxiety after a single exposure of inescapable footshocks (Chen et al., 2014). The increase in mRNA lasted for a couple of weeks and appeared to be related to an arousal-related increase in orexin neuron activity (Chen et al., 2014). Our group also reported that systemic injections of OX1R or OX2R antagonists as well as a non-specific orexin antagonist in shocked rats reduced contextual fear and the avoidance tendencies that resulted from exposing rodents to inescapable footshocks (Chen et al., 2014; Wang et al., 2017). There is also evidence that the orexin system is critical for the expression of the autonomic and behavioral changes associated with a CO_2_-panic provocation model of panic anxiety (Bonaventure et al., 2017). Another group has shown that administration of the OXA peptide in the cerebral ventricles elicits anxiety-like behaviors in both mice and rats (Suzuki et al., 2005). There is also preclinical and clinical evidence that an enhanced level of orexin activity may contribute to the higher incidence of anxiety in females (Grafe and Bhatnagar, 2020).

The areas of the brain where the orexin peptides or antagonists act to modulate anxiety and fear remained largely unexplored until recently. Orexin fibers and receptors are found in many of the regions of the anxiety network including the BSTDL and CeL (Peyron et al., 1998; Marcus et al., 2001) and administrations of orexins in these areas of the cEA were reported to produce anxiety-like responses (Lungwitz et al., 2012; Avolio et al., 2014). The PVT contains a relatively high density of orexin fibers compared to what is present in the BSTDL and CeL (Li et al., 2011) and orexin fibers make putative synaptic contacts with neurons that project to the NAcSh (Kirouac et al., 2005; Parsons et al., 2006). These anatomical observations led us to postulate that orexins could modulate anxiety by acting on PVT neurons that innervate the NAcSh and the rest of the cEA (Li and Kirouac, 2008). In a series of investigations, our research group investigated if orexins act at the PVT to modulate anxiety-like behaviors in rats. First, we found that injections of the OXA and OXB peptides in the pPVT region decreased locomotor activity, increased bouts of immobility and avoidance of the center of an open field (Li et al., 2009, 2010a). In another study, we found that injections of the orexin peptides in the pPVT resulted in avoidance of the open arms and increased ethological behaviors in the EPM indicative of an anxiety state (Li et al., 2010b). In contrast to these findings, injections of GABA agonists in the pPVT was reported to decrease the time spent in the open arm of the EPM (Barson and Leibowitz, 2015) indicating that the PVT’s effect on anxiety may be complex and involves multiple neurotransmitters or neuromodulators. We speculated that activation of orexin receptors in the PVT enhances the saliency of threats (e.g., open spaces, novel objects, and bright lights). To further establish that endogenously released orexins modulated anxiety by acting at the PVT, our research group demonstrated that administrations of a specific OX2R antagonist in the pPVT attenuate anxiety-like behaviors in rats that had received footshocks 24 h prior to the EPM test (Li et al., 2010a). It is notable that the anxiolytic effects of the orexin antagonist were only observed in rats that had been previously shocked indicating that anxiogenic effects of orexins are only present in rats exposed to an acute fear-inducing situation. While the pPVT is involved in conditioned fear to discrete auditory cues (Li et al., 2014b; Do-Monte et al., 2015; Penzo et al., 2015), administration of an non-specific orexin antagonist in the pPVT during the fear expression test has no effect on freezing to conditioned tones (Dong et al., 2015). Interestingly, contextual fear expression was also not affected by blocking of orexin receptors in the pPVT while the same treatment decreased social avoidance and anxiety-like responses in the open field (Dong et al., 2015).

In summary, stress and anxiety are complementary states that engage many of the same neural circuits (Bystritsky and Kronemyer, 2014). Orexin neurons are more active under conditions of high arousal including exposure to stressful and aversive situations. High levels of arousal are likely to activate stress-responsive areas of the brain and promote anxiety by increasing the saliency of emotionally relevant cues including potential threats (Mahler et al., 2014). The effect of an acute but intense stress event on orexin neurons has been shown to last for days and up to several weeks (Chen et al., 2014). It is also noteworthy that the actions of orexins on PVT neurons in response to stressful situations or challenges may lead to neuroplastic changes that may make the PVT more sensitive to novel challenges (Heydendael et al., 2011). Accordingly, stress may make orexin neurons more responsive to arousing conditions leading to an enhanced sensitivity of the PVT neurons to novelty and potential threats. The arousal- and threat-related signals may increase the activity of PVT neurons that relay this amplified signal to NAcSh, BSTDL, and CeL.

## Neural Pathway for PVT Modulation of Anxiety

**FIGURE 2:**
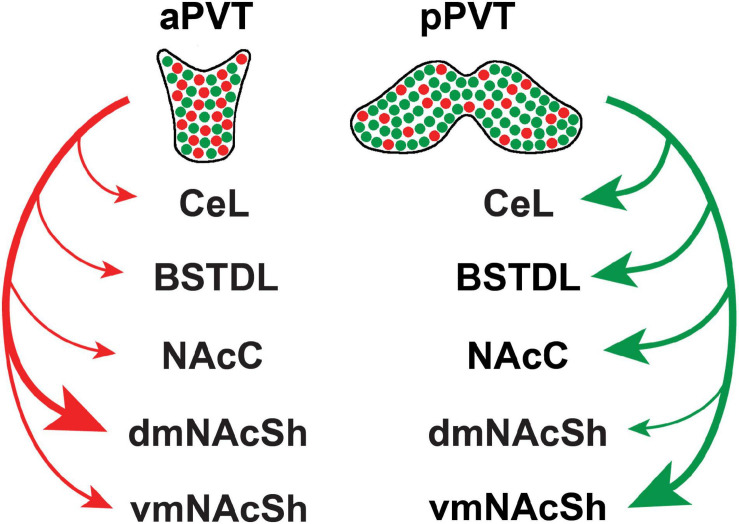
Summary of the efferent projections of the PVT based on recent retrograde tracing experiments involving various combinations of injections of the tracer in subcortical targets of the PVT (Dong et al., 2017). Projections appear to originate from two major population of intermixed neurons that preferentially innervate the dmNAcSh (red) and vmNAcSh (green) along with their collateral projections to other subcortical regions. The size of the arrow is indicative of the strength of the projection based on the number of retrograde-labeled neurons in the PVT from injections of cholera toxin B in the central nucleus of the amygdala (CeL), dorsolateral region of the bed nucleus of the stria terminalis (BSTDL), core of the nucleus accumbens (NacC), dorsomedial aspect of the shell of the nucleus accumbens (dmNAcSh), and ventromedial aspect of the shell of the nucleus accumbens (vmNAcSh). aPVT, anterior aspect of the paraventricular nucleus of the thalamus; pPVT, posterior aspect of the paraventricular nucleus of the thalamus.

There is experimental evidence that the PVT mediates freezing to conditioned tones as well as the immediate anxiogenic effects of footshocks (Li et al., 2014b; Do-Monte et al., 2015; Penzo et al., 2015; Pliota et al., 2018) via a projection to the CeL (Do-Monte et al., 2015; Penzo et al., 2015; Pliota et al., 2018). The pPVT may have a greater influence on fear and anxiety because this region of the PVT projects densely to the BSTDL and CeL (Li and Kirouac, 2008). There is an implicit assumption that a subpopulation of projection-specific neurons in the pPVT may mediate the defensive responses. For example, a PVT-CeL projection may mediate the behavioral freezing link to fear, whereas projections to the NAcSh or BSTDL may mediate the avoidance induced by potential threats. However, the idea that subpopulations of projection-specific neurons mediate unique defensive responses may be an oversimplification because recent anatomical evidence shows that PVT neurons have axons that bifurcate to innervate multiple targets (Unzai et al., 2015; Dong et al., 2017). Indeed, a detailed analysis and mapping of projection neurons in the PVT revealed that most neurons in the PVT innervate the NAcSh and that many of these neurons issue collaterals to the BSTDL and CeL (Dong et al., 2017). Neurons that project to the NAcSh, BSTDL, and CeL are intermixed throughout the aPVT and pPVT and do not form clusters of unique subpopulations of projection specific neurons. One caveat to this statement is that neurons that innervate the core of the nucleus accumbens and the ventromedial region of the shell (vmNAcSh) are located slightly more dorsally and laterally in the PVT, respectively, than those innervating the dorsomedial region of the shell (dmNAcSh). However, there are some notable differences in terms of the number of neurons in the aPVT and pPVT that project to various subcortical regions. As shown in Figure 2, neurons that project to the vmNAcSh and core of the nucleus accumbens are more likely to originate from the pPVT. These pPVT neurons are also more likely to send collaterals that innervate the BSTDL and CeL. In contrast, neurons that innervate the dmNAcSh are more likely to originate in the aPVT and are less likely to project to the BSTDL and CeL. This points to the possibility that PVT neurons that innervate the vmNAcSh along with their collaterals to the BSTDL and CeL may form a projection system that may be involved in mediating aversive or defensive responses. Our group recently tested this hypothesis by examining if chemogenetic inhibition of PVT neurons that project to the vmNAcSh interfered with the lasting behavioral changes produced by exposing rats to a single episode of inescapable foothocks (Dong et al., 2020). An intersectional chemogenetic approach was used to demonstrate that inhibition of PVT neurons that project to the vmNAcSh attenuates the lasting social avoidance that develops following exposure of rats to footshock stress. Interestingly, anxiety-like behaviors in the open field and contextual fear expression were unaffected by the same manipulation. Evidence that the projection to the vmNAcSh was involved was provided by showing that injections in the vmNAcSh of the agonist for a designer receptor exclusively activated by a designer drug (DREADD) had the same effect as systemic injections of the agonist. Furthermore, expression of the immediate early gene cFos was use to show that these effects were mediated by neurons in the NAcSh that contain the opioid peptide dynorphin (Dong et al., 2020). Dynorphin containing medium spiny neurons in the NAcSh have been shown to mediate the aversive effects of stress on behavior (Newton et al., 2002; Barrot et al., 2005; Bruchas et al., 2008; Land et al., 2008; Al-Hasani et al., 2015). The exact region of the NAcSh critical for social avoidance remains unknown because the intersectional DREADDs approach resulted in fibers being transduced in much of the medial NAcSh (Dong et al., 2020). Another unresolved question is whether the anxiety-like behaviors in the open field and/or the freezing associated with contextual fear expression are mediated by fiber collaterals to the BSTDL and CeL that originate from the same PVT-vmNAcSh projection neurons that mediate social avoidance. Indeed, PVT-NAcSh projecting neurons that contribute to social avoidance could also mediate decreases in exploratory behavior and freezing via collaterals to the BSTDL and CeL depending on the situational factors present during the test condition (i.e., presence of a social target, open areas, or shock context). A population of PVT neurons that send divergent projections to the NAcSh, BSTDL, and CeL could provide signals that help coordinate the selection or expression of different defensive responses.

## Model for How the PVT Contributes to Anxiety

Neurons in the NAcSh and cEA integrate signals from a number of sources including the thalamus, cortex and other areas of the brain resulting in the selection and expression of appropriate behavioral responses via activation of multisynaptic descending pathways (Pennartz et al., 1994; Cardinal et al., 2002; Zahm, 2006; Nicola, 2007; Humphries and Prescott, 2010; Floresco, 2015). This canonical view of the corticostriatal circuits posits that striatal neurons integrate signals and select appropriate responses based on previous learning contingencies and the behavioral state of the organism. An intriguing possibility is that the PVT’s divergent projections could contribute to different types of defensive responses based on situational factors present as well as the emotional or behavioral state of the organism. For example, activation of PVT fibers in the NAcSh could promote social avoidance if a social contact is present, activation of fibers to the BSTDL could support avoidance of open spaces and decrease foraging behavior, whereas activation of PVT fibers to the CeL could support freezing to cues and contexts previously associated with an aversive event. We know from studies using the expression of cFos that PVT neurons are active during states of high arousal including exposure to stressful/aversive conditions and presentation of cues/contexts signaling potential threats (Silveira et al., 2001; Linden et al., 2003, 2005; Salome et al., 2004; Hale et al., 2008; Galvis-Alonso et al., 2010; Kirouac, 2015). As shown in Figure 1, the PVT receives afferents from a number of brain regions involved in arousal and threat detection. Prefrontal cortical areas may relay threat-related signals to the PVT since these cortical areas have been shown to be involved in contextual fear, fear generalization and anxiety (Bermudez-Rattoni et al., 1997; Corcoran and Quirk, 2007; Laurent and Westbrook, 2008; Biedenkapp and Rudy, 2009; Stevenson, 2011; Alves et al., 2013; Kheirbek et al., 2013; Jiang et al., 2014; Rozeske et al., 2015; Wang et al., 2015; Zhang et al., 2015). The prelimbic cortex may be especially critical since it is activated by anxiogenic conditions (Linden et al., 2003, 2005; Hale et al., 2008; Wall et al., 2012) and has been shown to play a role in generating anxiety-like responses (Jinks and McGregor, 1997; Lacroix et al., 2000; Sullivan and Gratton, 2002; Shah and Treit, 2003, 2004; Lisboa et al., 2010; Yamada et al., 2015; Suzuki et al., 2016; Shimizu et al., 2018). The dorsomedial nucleus of the hypothalamus projects significantly to the PVT (Thompson et al., 1996; Li and Kirouac, 2012) and is another potential source of anxiety-related signals because activation of this hypothalamic nucleus has been shown to generate panic and anxiety (Fontes et al., 2011; Johnson and Shekhar, 2012). Finally, the PVT contains a variety of peptidergic fibers that originate from neurons in the hypothalamus and brainstem (Freedman and Cassell, 1994; Otake and Nakamura, 1995; Kirouac et al., 2005, 2006; Otake, 2005). These peptides could signal emotional and behavioral states similar to what has been shown for the orexins (Li et al., 2010a, b; Dong et al., 2015).

## Summary and Future Directions

The model emphasizes the hypothesis that the PVT integrates top-down signals related to potential threats with bottom-up signals related to emotional and behavioral states to energize defensive responses by activating descending pathways in the NAcSh and cEA. Cortical areas where the memory of aversive experience is processed and stored would provide the key signals that trigger striatal neurons to generate defensive response. The model advances the view that the PVT receives and integrates threat-related signals from the cortex along with behavioral or emotional state signals from the hypothalamus and brainstem. In this model, the PVT serves to integrate threat and situational information in a way that promotes appropriate defensive responses via its divergent projections to the NAcSh and cEA. The model also proposes that the PVT serves to promote or amplify the influence of the cortex on subcortical regions. The proposed model is focused on how the PVT regulates defensive behaviors. Nonetheless, the model is also pertinent for understanding how the PVT mediates appetitive behaviors. For example, recent evidence shows that signals from orexin and prelimbic cortical neurons converge and act at the PVT to modulate reward seeking responses to cues in a manner similar to what is predicted by the model (Otis et al., 2017, 2019; Campus et al., 2019).

Going forward it will be important to design experiments in which the contribution of the PVT on complex behavior can be examined in experimental situations where both appetitive and aversive outcomes are possible as recently done by some research groups (Do-Monte et al., 2015; Zhu et al., 2018; Choi et al., 2019). It would also be of interest to know if neurons in the PVT affect the behavior produced in the Vogel or the Geller and Seifter conflict tests of anxiety where rodents are punished by electrical shocks when trying to consume food or water (Millan, 2003). It will also be essential for future studies to consider how simultaneous activation of PVT fibers to multiple subcortical target regions affects behavior. This could involve determining how synchronized modulation of different collateral terminal sites affects behavioral responses driven by complex contingencies or behavioral states. A combination of opto- and chemogenetic approaches might be useful despite the technical challenges involved. It will also be of importance to determine how cortical inputs interact with PVT inputs at subcortical levels. For example, are signals from the prefrontal cortex amplified by the PVT in a manner that enhances the threat response driven by activity from the prefrontal cortex to the NAcSh, BSTDL, and CeL? It is clear that there are many challenges in studying the PVT especially when we consider the complexity of the PVT’s connections and the multitude of factors that modulate behavior.

## Author Contributions

The author confirms being the sole contributor of this work and has approved it for publication.

## Conflict of Interest

The author declares that the research was conducted in the absence of any commercial or financial relationships that could be construed as a potential conflict of interest.
